# How Often Are Ineffective Interventions Still Used in Clinical Practice? A Cross-Sectional Survey of 6,272 Clinicians in China

**DOI:** 10.1371/journal.pone.0052159

**Published:** 2013-03-22

**Authors:** Xiao-Min Luo, Jin-Ling Tang, Yong-Hua Hu, Li-Ming Li, Yan-Ling Wang, Wei-Zhong Wang, Li Yang, Xiao-hui Ouyang, Guang-cai Duan

**Affiliations:** 1 Center for Evidence Based Medicine, Peking University Health Science Centre, Peking University, Beijing, China; 2 Division of Epidemiology, School of Public Health and Primary Care, The Chinese University of Hong Kong, Hong Kong SAR, China; 3 School of Public Health, Peking University Health Science Centre, Peking University, Beijing, China; 4 Institute of Pathogen Biology, The Chinese Academy of Medical Sciences and Peking Union Medical College, Beijing, China; 5 School of Public Health, Guangxi Medical University, Nanning, Guangxi Zhuang Autonomous Region, China; 6 Inner Mongolia Medical College, Hohhot, Inner Mongolia, China; 7 Henan University of Science and Technology, Luoyang, Henan Province, China; Chancellor College, University of Malawi, Malawi

## Abstract

**Background:**

The World Health Organization reported in 2011that irrational use of medicines was a serious global problem that is wasteful and harmful. The worst is use of ineffective or harmful interventions which should not be used at all. However, little is known about the changes that 20 years of evidence-based medicine has made particularly in reducing use of ineffective interventions. We surveyed clinicians in China to show how often ineffective interventions were still used in practice.

**Methods:**

3,246 clinicians from 24 tertiary hospitals were surveyed in person and another 3,063 through an online survey between 2006–2007. The main outcomes are prescription by a clinician, and use in a patient of, an ineffective intervention and of a matched effective intervention in patients with the same disease. 129 ineffective interventions for 68 diseases were identified from the BMJ Clinical Evidence and included in the survey. One effective intervention was identified for each disease and a total of 68 effective interventions were thus also included. The frequency of use of effective interventions was used as a reference for that of ineffective intervention.

**Results:**

The mean prescription rate by clinicians is 59.0% (95% confidence interval (95% CI): 58.6% to 59.4%) and 81.0% (95% CI: 80.6% to 81.4%) respectively for ineffective and effective interventions. The mean frequency of use in patients is 31.2% (95% CI: 30.8% to 31.6%) and 56.4% (95% CI: 56.0% to 56.8%) for ineffective and effective interventions respectively. The relative reduction in use of ineffective interventions as compared with that of matched effective interventions is 27.2% (95% CI: 27.0% to 27.4%) and 44.7% (95% CI: 44.3% to 45.1%) for clinician's prescription and use in patients respectively. 8.6% ineffective interventions were still routinely used in practice.

**Conclusions:**

Ineffective interventions were still commonly used. Efforts are necessary to further reduce and eventually eliminate ineffective interventions from practice.

## Introduction

In 1992, a seminal paper showed clinical practice could have a time lag of 10 or more years behind what the evidence would suggest [1]. In the same year, evidence based medicine was proposed and urged medical decisions to be made consistent with current best evidence [2], [3], [4], [5], [6]. In the past 20 years, tremendous efforts, in particular in generating, synthesizing and delivering evidence, have been made to help and engage clinicians and policy makers in evidence-based decision making [7], [8], [9], [10].

It has been shown beyond reasonable doubt that many widely used interventions are ineffective and should be eliminated from practice [11]. However, little is known whether 20 years of efforts in evidence-based medicine have made any difference in clinical practice in particular in developing countries where healthcare resources are sparse. We thus conducted a cross-sectional survey of 6,272 clinicians in China to show how often ineffective interventions are still used in practice.

## Methods

The objective of the study is to identify and compare the frequencies of using ineffective interventions. Clinical decision on an ineffective intervention can be made primarily according to evidence: ineffective interventions deliver no benefit but harm to patients, waste resources and thus should not be used at all. Thus, any use of ineffective interventions if found can be considered inappropriate. The frequencies of using different ineffective interventions are, however, not directly comparable as they may not be used in all patients. Even if they were effective, a low frequency of use does not necessarily mean an intentionally reduced usage. In order to make meaningful comparisons among ineffective interventions, a matched effective intervention for the same disease was indentified and the frequency of use of the effective intervention can provide a necessary reference for interpreting the use of the ineffective intervention.

### Identification of Ineffective and Reference Effective Interventions

#### Ineffective Interventions

By ineffective, we refer to therapies that are either not beneficial or can do more harm than good. According to the 15^th^ edition of the *BMJ Clinical Evidence*, one of the most authoritative sources of clinical evidence, we identified 129 interventions which were shown to be ineffective as compared with a placebo or no intervention, from 196 interventions that are classified in Clinical Evidence as either “unlikely to be effective” or “likely to ineffective or harmful” [11]. They are related to 68 diseases.

#### Effective Interventions

For each of the 68 diseases, one effective intervention was identified and included in the survey as a reference for the ineffective intervention(s). Matched effective interventions were used in parallel to the ineffective ones with a view to reducing possible biases which may arise if clinicians knew use of ineffective interventions was investigated.

A matched effective intervention was either randomly selected from interventions shown to be effective in Clinical Evidence for the same clinical problem as compared with a placebo or no intervention, or from the first-line interventions recommended by national clinical textbooks in China. Forty-eight effective interventions were identified from effective or likely to be effective categories in the Clinical Evidence and 20 from interventions recommended in Chinese textbooks. A list of the 129 ineffective interventions and 68 matched effective interventions including the related disease, the outcome the intervention is to favorably modify, and the department(s) in which the intervention is mostly used is shown in Table S1.

#### Translating Names of Interventions

The interventions, diseases, and outcomes were translated according to Chinese clinical textbooks supplemented by the widely used Pharmacopoeia in the country [12]. Two experienced clinicians in each of the 17 clinical disciplines were invited from Peking University Health Science Center to validate and finalize the translation.

### Design and Validation of the Questionnaire

Data on the frequency of use of ineffective and effective interventions were all collected with a questionnaire designed specifically for this study. The questionnaire contained primarily a simple question about the use of an ineffective intervention and of the matched effective intervention, which was repeatedly asked for all the ineffective and effective interventions. The question had four requisite components: the intervention, disease, outcome, and frequency of using the intervention in patients with the disease in the past 12 months. Each question had two sub-questions. The first sub-question was, for example, “In the past 12 months, had you ever used bed-rest to help relieve pain and disability in patients with acute low back pain?” Three response options were allowed: yes, no, and never heard of the intervention. The third option “never heard of the intervention” was in fact very rare and also meant “never used”. Therefore the results of second and third options were combined in data analysis. If the answer was yes, the clinician would be further asked in the second sub-question to estimate how often he/she had used the intervention to reduce pain and disability in every 10 patients with acute low back pain in the past 12 months. Details are included in Text S1.

Based on the answers to the two questions, two major indexes to describe the frequency of using a specific intervention can be quantified:

1. The prescription rate of clinicians, defined as the percentage of clinicians who ever prescribed the concerned intervention in the past 12 months to patients with a certain clinical condition for achieving a specific objective (or outcome) among all clinicians who ever treated the same kind of patients with any methods during the same period. In computation, it is the number of clinicians who did not give a zero percentage in the second sub-question divided by all those who answered yes to the first sub-question (Text S1).
2. The frequency of use in patients, is the percentage of patients with a certain disease and treated by clinicians in the past 12 months with the concerned intervention for achieving a specific objective among all patients with the same disease and treated by the same clinicians for the same objective but with any therapeutic methods during the same period. In computation, it is the sum of the frequency stated in the second sub-question divided by the total number of clinicians who answered yes to the first sub-question.

Other data collected included gender, age, number of years of clinical experience, education, professional title, type of hospital and location of hospital. Interventions were divided into 17 categories according to the clinical department in which they were mostly used.

The initial questionnaire was revised three times following two pilot studies and one panel discussion of senior clinicians and epidemiologists. In the first pilot study, face to face interviews were conducted in 45 clinicians from 13 departments of a tertiary hospital in Beijing. Then a panel discussion of nine senior clinicians and two epidemiologists was organized to assess the content and structure validity of the revised questionnaire and the allocation of the department for the interventions. After the second revision, the questionnaire was tested in 28 clinicians in one of the hospitals where the survey was eventually conducted. In every revision, major changes were concentrated on refining and modification of the translation of the interventions, diseases and outcomes, improvement in asking the question on the frequency of use, and changes in department allocation of interventions.

In order to survey through the internet, the above questionnaire was uploaded to an online questionnaire system based on a browser/server mode designed by computer experts from the Institute of Computer Technology of the Chinese Academy of Sciences. After answering the questions on general information, a question on an intervention classified into the clinical department of the clinician would be randomly chosen by the computer. The doctor could stop the survey anytime or continue until all the questions relevant to his department were answered. Only those who had answered questions on general information and at least one relevant question on interventions were included in the analysis.

### Selection of Clinicians and Administration of Questionnaire

The survey has two parts: a field survey and an online survey through the internet. In order to reduce biases, in both surveys clinicians were told that the study was to estimate the frequency of use of various interventions rather than investigating into ineffective interventions. The letters to clinicians in the field survey and the online survey are provided in Text S2 and Text S3 respectively. Thus, all the surveyed clinicians and those involved in organizing the field work were not told that ineffective interventions were included in the questionnaire.

In mainland China, hospitals are graded into three levels according to the standard and quality of service. Level 3 hospitals are the best and mostly are the major hospitals in large cities. For the field survey, 24 level 3 hospitals (including 14 general hospitals and 10 specialized one) in 3 medium-sized cities in different provinces were selected. The three cities were deliberately selected from northern, central and southern parts of China representing the country's average economic, medical and educational status in the country. All clinicians currently working in one of the following 17 departments were invited to participate: cardiology, dentistry, ENT (ear-nose-throat), gastroenterology, general surgery, infectious diseases, neurology, neurosurgery, nephrology, respiratory, obstetrics and gynecology, oncology, ophthalmology, orthopedics, pediatrics, psychiatry, and sexually transmitted diseases and dermatology. Those who were eligible for the survey had treated at least one patient with one of the 68 diseases in the past 12 months. Clinicians of a department received a questionnaire containing only interventions related to that department.

The field survey was conducted between July and November 2006. Through the hospital's central administration and the head of department, we obtained the full list of clinicians in a department, including visiting clinicians and those on leave such as maternal leave. The survey was mostly conducted in the department at a time convenient for clinicians. A written informed consent (Text S2 and Text S3) was obtained from the clinician before the questionnaire was self-administered under the observation of a trained interviewer. A clinician was considered non-respondent after a maximum of three failed interview visits.

The online questionnaire was made accessible for 9 months from December 2007 from the websites of Lilac Garden (DXY), Good Doctors, and Peking University EBM Centre, which altogether could allow 180 people to answer the questionnaire simultaneously. There are 4 million and 2.0 million people who have registered with DXY and Good Doctors websites respectively with most being health professionals. After the questionnaire was made available, an advertisement was publicized in the front page of the 3 websites. The detailed steps of the online survey are available (Figure S1). Over 3,000 clinicians responded from various hospitals in 31 provinces and municipalities. One month after completion of the online survey, a reproducibility study was conducted by inviting 85 clinicians to re-answer the same questions. As planned for the field survey and anticipated for the online survey, the majority of surveyed clinicians would be from level 3 hospitals as it is our objective to make an inference that the national average situation is likely to be worse than what we may find.

We assumed on average the effective intervention would be used in 50% of patients and there would be 20% absolute reduction in the use of ineffective intervention. Further assuming type I error to be 0.05 and a statistical power of 80%, 100 clinicians would be required for each pair of intervention. For the 129 ineffective interventions, 12,900 clinicians would be required. Considering one clinician may be able to answer the questions on over half of the ineffective interventions commonly used in his department which is approximately 4, the required sample size will be around 3,200.

### Data Management and Statistical Analysis

Data from the field survey were double entered and compared for consistency with Epidata 3.0. The online survey data was automatically stored by Access and can be analyzed directly. Data validity was also checked. For example, we excluded clinicians who reported data with the same frequency (such as 0% or 100%) for all interventions and online surveyed clinicians who registered the survey more than once with the same name, IP and email. SPSS 15.0 was used for merging the two data sets and for statistical analyses.

Clinicians surveyed through the two methods were compared in education, clinical experience, hospital, and demographic data. The main result is the prescription rate of clinicians and the frequency of use in patients. Mean, 25^th^ centile, median and 75^th^ centile were used to summarize the two parameters. As the number of clinicians who answered questions on different interventions varied, a weighted average was also computed wherever deemed applicable. As not every effective intervention is used in every (or 100%) eligible patient, an absolute reduction from 5% to 0%, is not the same as that from 100% to 95%. For the former, the ineffective intervention is completely stopped, while for the latter the ineffective intervention is still used almost routinely, the waste remains similar and a great effort is needed in order to stop it. The relative reduction will reflect such different situations. We thus also defined a relative reduction in the frequency of use in patients (RR-PU), which is the difference in the frequency of use between effective and ineffective interventions relative to the former. RR-PU gives an idea by how much an ineffective intervention has been made less used as compared to the hypothetical prescription rate with which it could be used if it were effective. The relative reduction in prescription rate of clinicians is similarly defined and interpreted.

According to the shape of the distribution of the frequency of use in patients, interventions could be divided into 4 categories: left skewed, right-skewed, U-shaped, and uniformly distributed. Some are highly skewed to the left (never used) and with a median below 20%, implying for a clear “consensus” that the intervention should be stopped. But this consistency between evidence and practice could partly due to the fact that the intervention was new and many clinicians had not known it yet. Conversely, some were highly skewed to the right (always used) and with a median above 80%, implying for a clear consensus that the intervention should always be used. This is the worst situation in which evidence was totally ignored in practice. Some are U-shaped, implying clinicians are sharply divided according to the use of an intervention: some often used it and some rarely used it. Such interventions include all those with a median between 20% and 80%, with over 50% of clinicians stating a frequency of use between 0∼10% or between 90∼100%, and with a difference below 30% between the percentage of clinicians stating a frequency of between 0∼10% and that between 90∼100%. The rest are considered as uniformly distributed with a central mode or without an obvious mode, implying there was no good consensus whatsoever about the use of the intervention.

Differences in the weighted mean and median were tested by using the student t-test and the nonparametric Mann-Whitney test respectively. Categorical variables were tested with the Chi-squared test for variations among different comparison groups. The statistical significance level was all set at 0.05. We also used multiple logistic regression to explore factors that may influence the use of ineffective interventions.

## Results

### Characteristics of Subjects

The field questionnaire was sent to 3,370 clinicians and 3,246 satisfactorily completed the questionnaire, resulting in a response rate of 96%. A total of 4,635 clinicians logged in the online survey system and 3,026 (65%) satisfactorily answered the question for at least one intervention. The characteristics of the surveyed clinicians were described in Table 1.

**Table 1:** Characteristics of all 6,272 Surveyed Clinicians.

Characteristics	Field Survey (n = 3246)	Online Survey (n = 3026)	Total (n = 6272)
	Data below are either nnumber (%) or Mean±SD		
Males	n = 3,179	n = 2,951	n = 6,130
	1,780(56.0)	2,302(78.0)	4,082(66.6)
Age (years)	37.9±9.9	32.1±6.6	34.8±8.8
Years of clinical working experience	12.0±9.7	8.9±7.2	10.5±8.7
Medical education	n = 3,156	n = 3,026	n = 6,182
PhD or MD	167(5.3)	268(8.9)	435(7.0)
Master degree	895(28.4)	898(29.7)	1793(29.0)
MB	1,756(55.6)	1,373(45.4)	3,129(50.6)
Educations below MB	338(10.7)	487(16.1)	825(13.3)
Professional title	n = 3,195	n = 3,026	n = 6,221
Chief clinicians ( = consultant)	502(15.7)	121(4.0)	623(10.0)
Associate chief clinicians	660(20.7)	426(14.1)	1,086(17.5)
Attending clinicians	993(31.1)	1,129(37.3)	2,122(34.1)
Residents	1,040(32.6)	1,350(44.6)	2,390(38.4)
Level of hospital	n = 3,178	n = 3,026	n = 6,204
Level 3 (highest)	2,624(82.6)	1,556(51.4)	4,180(67.4)
Level 2	457(14.4)	1,053(30.8)	1,510(24.3)
Level 1	73(2.2)	417(13.8)	490(7.9)
Others (unclassifiable: lowest)	24(0.7)	0(0.0)	24(0.4)
Location of hospital	n = 3,191	n = 3,026	n = 6,217
Municipality (e.g. Beijing)	N/A	390(12.9)	390(6.3)
Capital city of a province	2,562(80.3)	727(24.0)	3,289(52.9)
Capital city of a prefecture	312(9.8)	1,005(33.2)	1317(21.2)
City of a county, town or below	317(9.9)	904(29.9)	1,221(19.6)

129 interventions for 68 different diseases and conditions were included in the survey. The three diseases with the largest number of ineffective interventions prescribed were acute renal failure (9 ineffective interventions), ischaemic cardiac events (7) and depression in children and adolescents (6) (Table S2). The distribution of clinicians' answers to a typical question is shown in Figure S2.

### Prescription rate of clinicians and frequency of use in patients

The distribution of prescription rate of clinicians and frequency of use in patients for all the effective and ineffective interventions was shown in Figure S3. The weighted mean, simple mean, median and quartiles for both ineffective and effective interventions are shown in Table 2 respectively for the prescription rate of clinicians and frequency of use in patients. For most interventions, the difference in both the rate of use in patients and prescription rate of clinicians between the ineffective and matched effective interventions is statistically significant (Table S1). Eleven ineffective interventions (sig-type = a) had a difference in both the rate of use in patients and the rate of prescription by clinicians, 7 (sig-type = b) in only the rate of prescription by clinicians and 3 (sig-type = c) in the rate of use in patients, between the ineffective and matched effective interventions that is statistically insignificant.

**Table 2:** Prescription rate by clinicians (%), frequency of use in patients (%) and their relative reduction: a summary for all 129 ineffective interventions and 68 effective interventions in 6,272 clinicians.

Summary Indexes		Prescription rate by clinicians (%)				Frequency of use in patients (%)			
		Ineffective Interventions (PIT* = 53,268)a	Effective Interventions (PIT = 27,465)b	AR-PR*c = b-a	RR-PR*d = (b-a)/b (%)	Ineffective Interventions (PIT = 53,268)e	Effective Interventions (PIT = 27,465)f	AR-PU*g = f-e	RR-PU*h = (f-e)/f (%)
Weighted Mean±SE		58.1±2.2	78.0±2.6	19.9±0.5	25.5±0.4	31.4±1.8	52.5±3.0	21.1±0.7	40.2±0.1
Simple Mean±SE		59.0±0.2	81.0±0.2	22.0±0.3	27.2±0.1	31.2±0.2	56.4±0.2	25.2±0.7	44.7±0.2
Percentiles	25th	37	69.5	32.5	46.8	12.2	31	18.8	60.6
	Median	63	86.5	23.5	27.2	27.4	49.7	22.3	44.9
	75th	80	95.0	15.0	15.8	42.5	71.4	28.9	40.5

Notes: PIT = clinician-intervention times; AR-PR = absolute reduction in prescription rate; RR-PR = relative reduction in prescription rate; AR-PU = absolute reduction in frequency of use in patients; RR-PU = relative reduction in frequency of use in patients.

**Figure 1:**
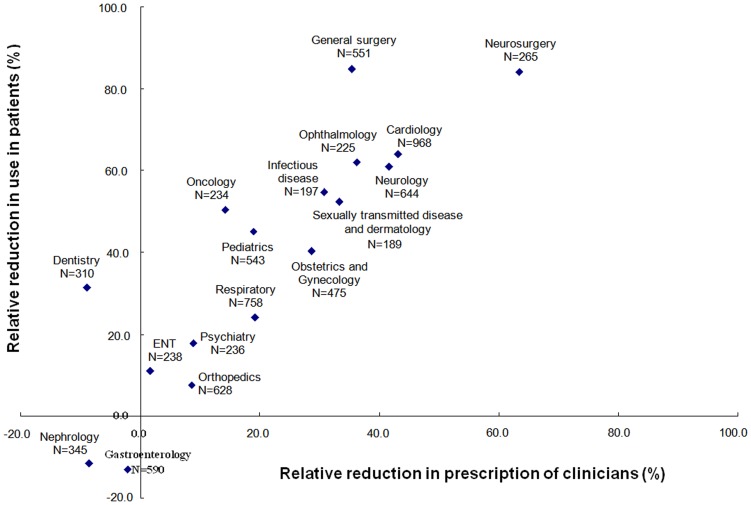
Relative reduction in use of ineffective interventions in the 17 departments.

There is a 27.2% reduction in the prescription of clinicians, and 44.7% in use in patients, of ineffective interventions relative to that of effective ones. The relative reduction in the 17 departments varies substantively with neurosurgery, general surgery and cardiology among the best and gastroenterology and nephrology among the worst (Figure 1). Detailed data are shown in Table S3.

The pattern of the distribution of the use of ineffective and effective interventions is summarized in Table 3. Although many more ineffective interventions (64.1%) are rarely used as compared to effective interventions (28.4%), 11 (or 8.6%) ineffective interventions are still routinely used. No good consensus (including U-shaped and uniformly distributed interventions) seemed to exist for a further 27.4% (35) of ineffective interventions, which is quite similar to 31.4% for effective interventions. The 11 routinely used ineffective interventions are:

**Table 3:** Number and percentage of interventions according to the four different patterns of use defined based on the frequency of use in patients.

Category	Ineffective interventions Total n = 128	Effective interventions Total n = 67	Relative Frequency (95% CI)
(1) Skewed to the left: rarely used	82 (64.1%)	19 (28.4%)	2.26(1.27–4.04)
(2) Skewed to the right: always used	11 (8.6%)	27 (40.3%)	0.21 (0.10–0.45)
(3) U-Shaped: practice sharply divided	7 (5.5%)	3 (4.5%)	1.22 (0.31–4.87)
(4) Uniformly distributed: no clear consensus	28 (21.9%)	18 (26.9%)	0.81(0.42–1.57)

Notes: one intervention with less than 15clinicians available was excluded from both the effective interventions and ineffective interventions.

1. bed rest for acute low back pain and sciatica to reduce pain and disability;
2. bed rest for herniated lumbar disc to reduce pain and disability and increase satisfaction;
3. routine preoperative traction for treat hip fracture to relieve preoperative pain or increase subsequent ease and quality of fracture reduction at time of surgery;
4. motility stimulants as initial intervention for gastro-esophageal reflux disease to relieve symptoms;
5. cold intervention for ankle sprain to relieve symptoms;
6. nitrates for acute myocardial infarction to reduce mortality;
7. antibiotics (oral) for otitis media with effusion to cure;
8. loop diuretics to prevent acute renal failure in high-risk patients;
9. non-steroidal anti-inflammatory drugs for herniated lumbar disc to increase overall improvement;
10. back exercises for acute low back pain and sciatica to reduce pain or disability;
11. magnesium sulphate for stopping contractions during preterm labour.

**Figure 2:**
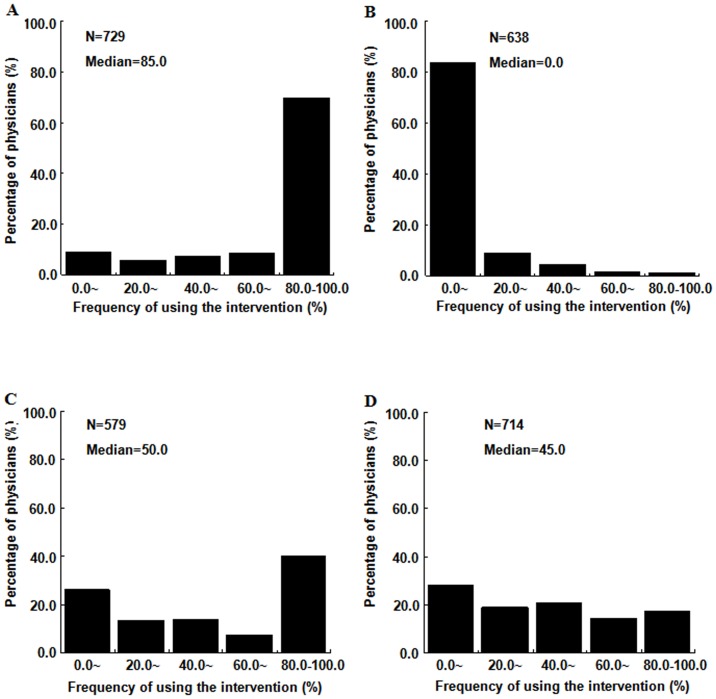
Examples of the four patterns of the distribution of the frequency of using ineffective interventions in patients. Panel A: Almost always used: Nitrates on top of thrombolysis in acute myocardial infarction to reduce the mortality; Panel B: Rarely used: Hormone replacement therapy in menopausal women for secondary prevention of cardiovascular events; Panel C: Use sharply divided: H pylori eradication in H pylori positive people with gastro-oesophageal reflux disease; Panel D: No consensus on use: Calcium channel blockers for secondary prevention of ischeamic cardiovascular events.

The 35 ineffective interventions for which there was no good practice consensus are highlighted in yellow in Table S1. Figure 2 shows typical examples for the 4 types of interventions.

Multivariate regression analysis (Table 4) showed that female sex, less education, junior clinician, lower level hospital, younger age, and more years of clinical experience clinicians are statistically significantly (P<0.05) related to more prescription of the ineffective interventions. Less education, higher level hospitals, and older age are statistically significantly related to less prescription of effective interventions. Thus, comparisons between ineffective and effective interventions suggest that female sex, less education, junior clinician, more years of clinical experience are likely predictors of prescribing more ineffective interventions.

**Table 4:** Multivariate regression analysis on factors which may be related to the frequency of patient's use of ineffective interventions: results of stepwise general linear regression.

	Effective interventions		Ineffective interventions	
	Odds ratio (95% CI)	P	Odds ratio (95% CI)	P
1. Male sex	0.97 (0.91–1.04)	0.452	0.85 (0.82–0.89)	<0.001
2. Less education	0.92 (0.87–0.97)	0.002	1.07 (1.03–1.10)	<0.001
3. Junior clinicians	0.96 (0.90–1.01)	0.129	1.06 (1.03–1.10)	<0.001
4. Lower level hospitals	1.12 (1.05–1.19)	0.001	1.10 (1.06–1.14)	<0.001
5. Age in years	0.98 (0.98–0.99)	<0.001	0.98 (0.98–0.99)	<0.001
6. Years of clinical experience	1.00 (0.99–1.01)	0.567	1.01 (1.00–1.01)	0.004

## Discussion

In the history of medicine, it is not uncommon that ineffective interventions were widely used in clinical practice. For example, Thalidomide, a drug commonly used for relieving pregnancy-induced nausea and vomiting can cause phocomelus [13]. Lidocaine for preventing ventricular arrhythmia after myocardial infarction may cause death [2], [4], [5].

A systematical review of evidence showed that over 30% of commonly used interventions in obstetrics and childbirth were harmful or of unknown effectiveness [3]. A more comprehensive collection of evidence showed that among 3,000 commonly used interventions, 10% were ineffective or even harmful [11].

According to the World Health Organization [14], irrational use of medicines is an extremely serious global problem that is wasteful and harmful. Nevertheless, more than 50% of all medicines are still incorrectly prescribed. The situation is worse in developing countries. The worst incorrect use is probably the use of ineffective or harmful interventions. This survey in China found that ineffective interventions were indeed less used but, in an absolute term, they were still used very often. Some were used in almost all eligible patients.

This study is not a random sample of all Chinese clinicians. However, given clinicians in tertiary hospitals are over-represented in the study, it is likely that the national average situation would be even worse if representative number of clinicians in community and township hospitals were included. Furthermore, patient's records could have been but were not used in this study as the outcome to achieve by an intervention was often not recorded and thus could not be studied. Furthermore, studies have also shown that results from clinicians' self stated prescription reflect quite faithfully what they actually do in practice [15], [16], [17].

Before we conclude, we would like to note that in some occasions the use of an ineffective intervention might be justified for its placebo effect. However, this use will only be justifiable when no effective interventions are available for a disease which we believe is rare or when effective interventions are not accessible or not affordable which might be common in particular in developing countries.

In conclusion, ineffective interventions that should not be used at all have indeed been less used. However, the reduction was moderate and many were still routinely used in practice. Efforts are necessary to further reduce and eventually eliminate the use of ineffective interventions from practice.

## Supporting Information

Figure S1 Guides and process of the online survey.
(TIF)

Figure S2 Distribution of physicians' answers to a question.
(TIF)

Figure S3 Distribution of rate of prescription of physicians and rate of use in patients for all interventions. Panel A: Distribution of prescription rate of physicians for 68 effective interventions and 129 ineffective interventions in 6,272 physicians; Panel B: Distribution of the frequency of use in patients for 68 effective interventions and 129 ineffective interventions in 6,272 physicians.
(TIF)

Table S1 Description of the 129 ineffective and 68 effective intervetions.
(XLS)

Table S2 Distribution of the 68 related diseases or conditions for the 129 ineffective interventions included in this survey.
(DOCX)

Table S3 Prescription rate of physicians, relative reduction in prescription rate, frequency of use in patients, and relative reduction in frequency of use according to departments where the interventions are mostly used.
(DOCX)

Text S1 An example question asked for effective and ineffective interventions.
(DOCX)

Text S2 The letter to the surveyed clinicians.
(DOCX)

Text S3 A survey on the use of some clinical interventions in China.
(DOCX)
