# Supplementary figures and images for: How Often Are Ineffective Interventions Still Used in Clinical Practice? A Cross-Sectional Survey of 6,272 Clinicians in China
Source: PLoS One. 2013 Mar 22;8(3):e52159. doi: 10.1371/journal.pone.0052159 (PMC3606390; doi:10.1371/journal.pone.0052159)

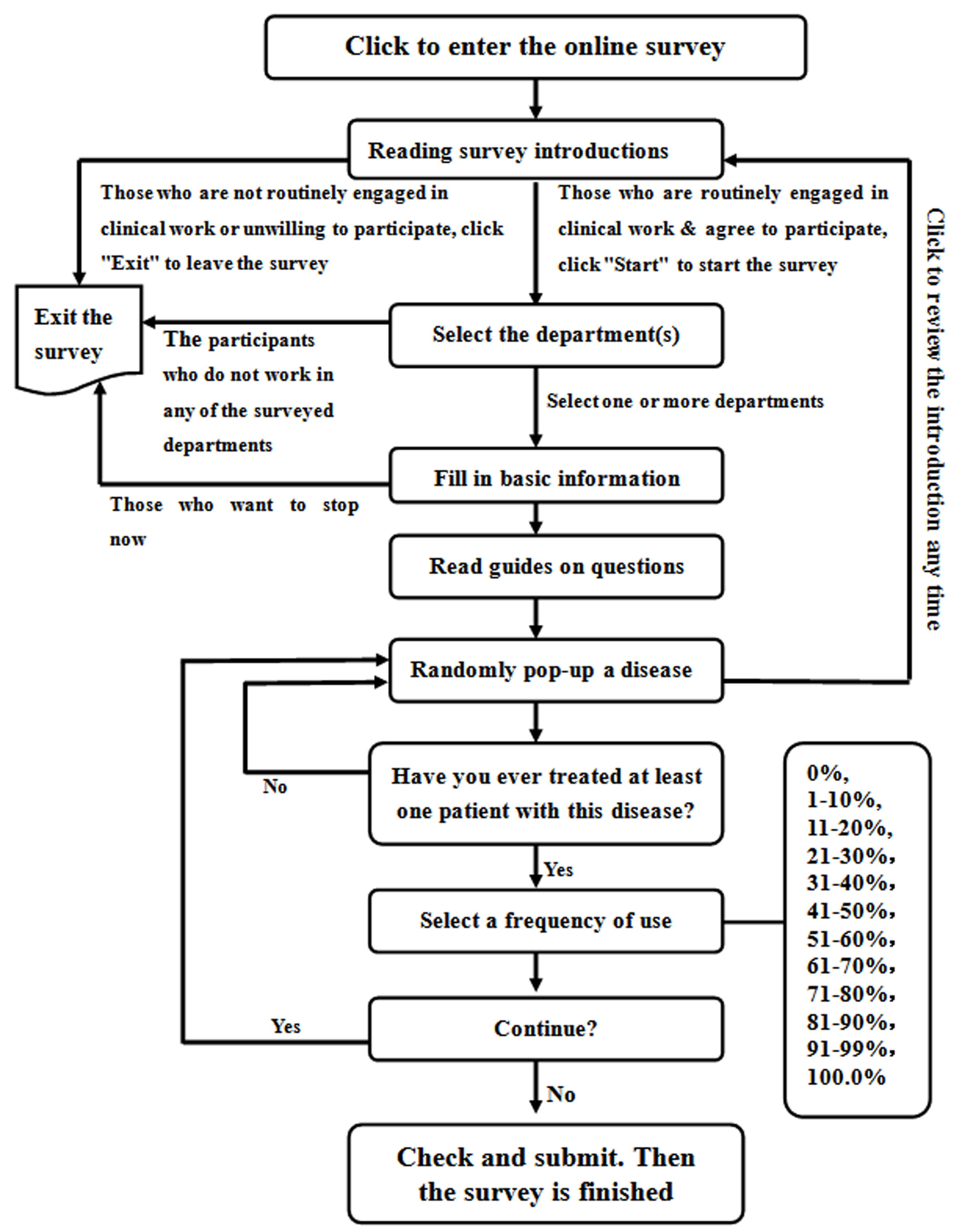

Supplement: Figure S1 — Guides and process of the online survey. (TIF) [file pone.0052159.s001.tif]

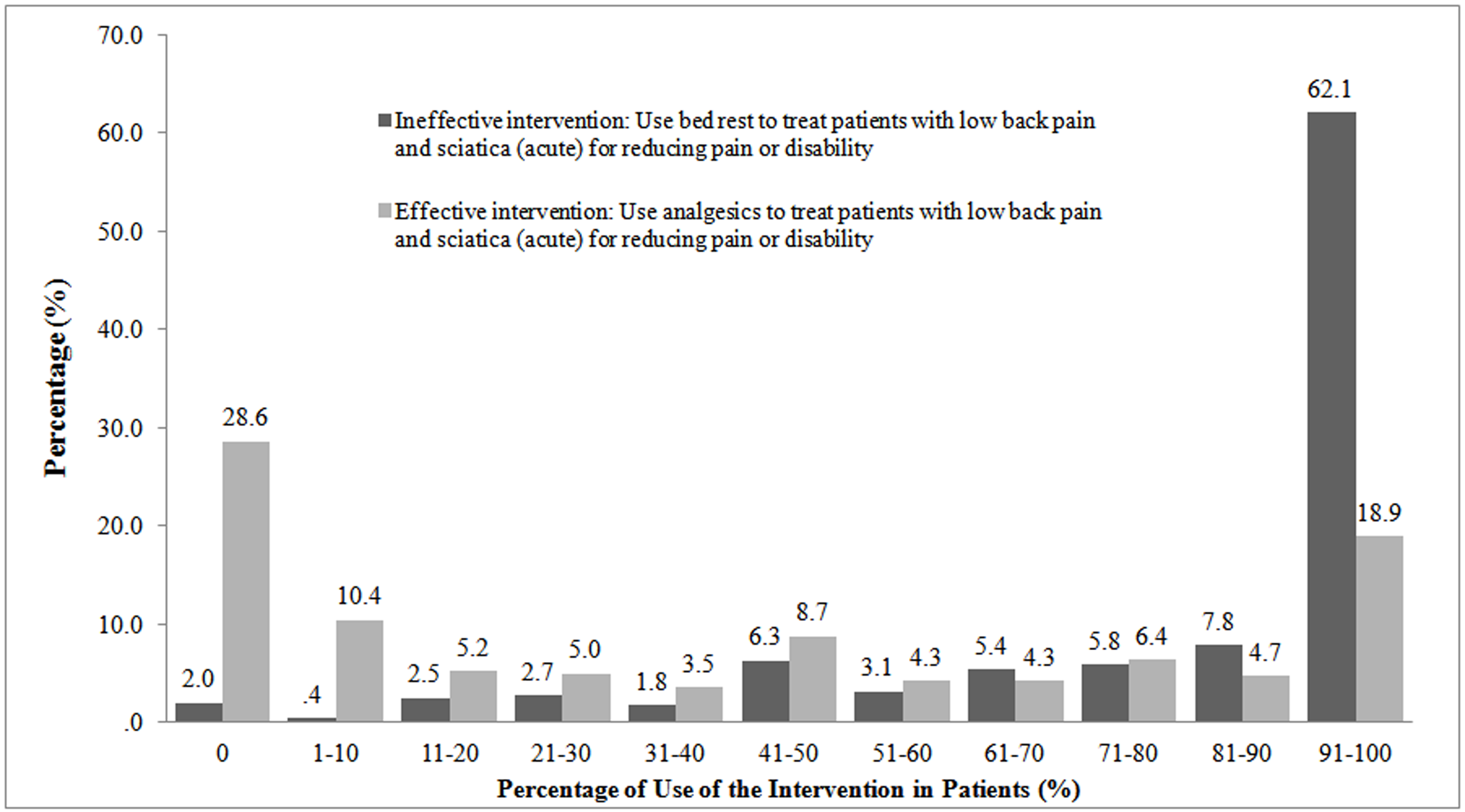

Supplement: Figure S2 — Distribution of physicians' answers to a question. (TIF) [file pone.0052159.s002.tif]

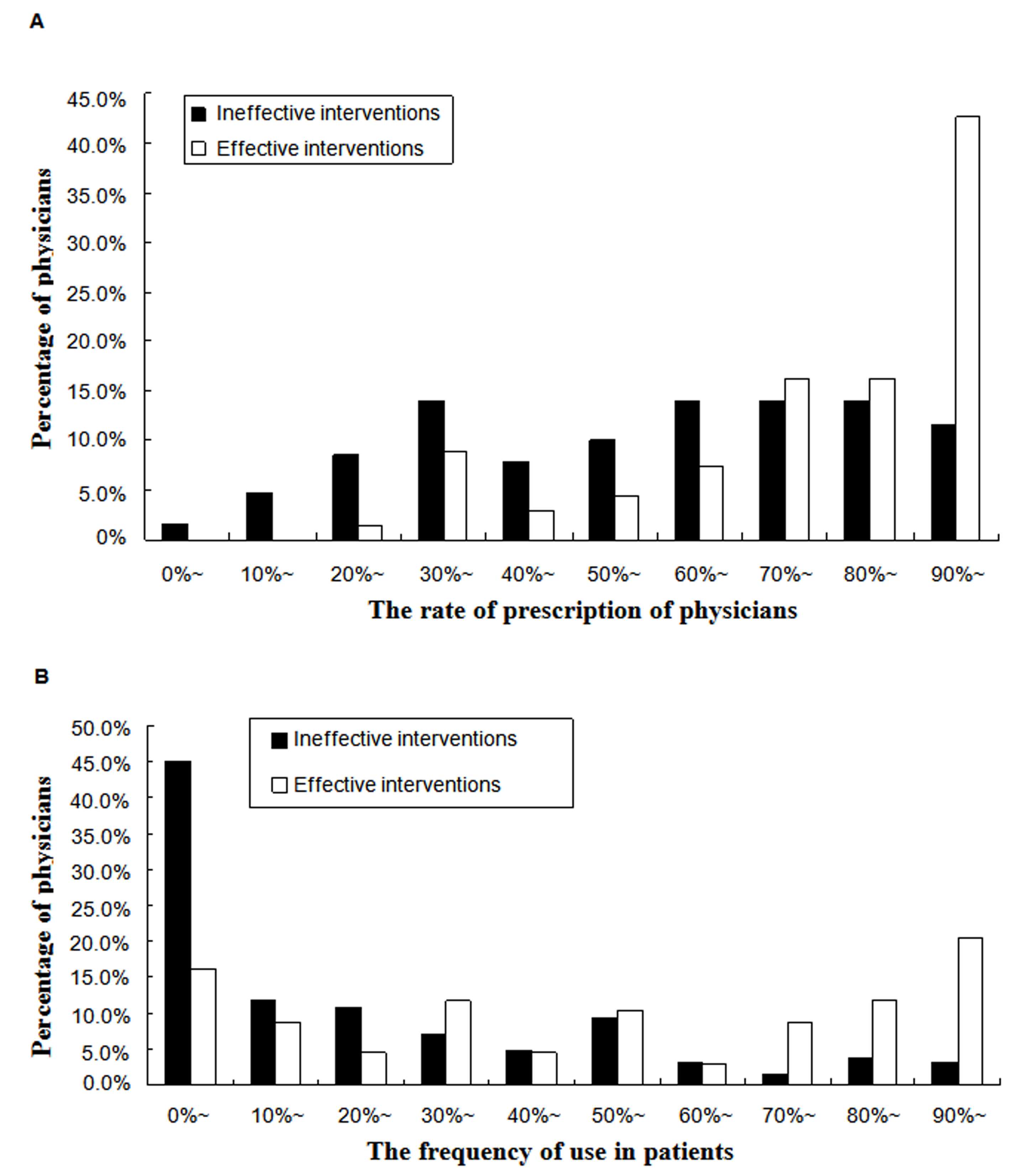

Supplement: Figure S3 — Distribution of rate of prescription of physicians and rate of use in patients for all interventions. Panel A: Distribution of prescription rate of physicians for 68 effective interventions and 129 ineffective interventions in 6,272 physicians; Panel B: Distribution of the frequency of use in patients for 68 effective interventions and 129 ineffective interventions in 6,272 physicians. (TIF) [file pone.0052159.s003.tif]
