# Supplementary material for: How Often Are Ineffective Interventions Still Used in Clinical Practice? A Cross-Sectional Survey of 6,272 Clinicians in China
Source: PLoS One. 2013 Mar 22;8(3):e52159. doi: 10.1371/journal.pone.0052159 (PMC3606390; doi:10.1371/journal.pone.0052159)
Supplement: Table S2 — Distribution of the 68 related diseases or conditions for the 129 ineffective interventions included in this survey. (DOCX) [file pone.0052159.s005.docx]

**Table S2. Distribution of the 68 related diseases or conditions for the 129 ineffective interventions included in this survey**

| **Related disease** | **Number of ineffective interventions included** |
| --- | --- |
| 1. Acute myocardial infarction | 3 |
| 1. Acute otitis media | 1 |
| 1. Acute renal failure (reducing mortality) | 1 |
| 1. Acute renal failure (preventing acute renal failure in people at high risk of acute renal failure) | 8 |
| 1. Age related macular degeneration | 2 |
| 1. Angina (unstable) | 1 |
| 1. Ankle sprain | 2 |
| 1. Anorexia nervosa | 1 |
| 1. Aphthous ulcers (recurrent) | 1 |
| 1. Atrial fibrillation (recent onset) | 1 |
| 1. Bacterial vaginosis | 2 |
| 1. Breast pain | 3 |
| 1. Candidiasis (vulvovaginal) | 3 |
| 1. Carpal tunnel syndrome | 2 |
| 1. Change behavior: smoking cessation | 2 |
| 1. Chronic fatigue syndrome | 1 |
| 1. Chronic obstructive pulmonary disease | 2 |
| 1. Common cold | 1 |
| 1. Community acquired pneumonia | 1 |
| 1. Dementia | 1 |
| 1. Depression in children and adolescents | 6 |
| 1. Domestic violence towards women | 1 |
| 1. Dysmenorrhoea | 1 |
| 1. Essential tremor | 1 |
| 1. Fracture prevention in postmenopausal women | 2 |
| 1. Gastro-oesophageal reflux disease | 3 |
| 1. Genital herpes | 1 |
| 1. Genital warts | 1 |
| 1. Headache (chronic tension-type) | 2 |
| 1. Heart failure | 3 |
| 1. Herniated lumbar disc | 3 |
| 1. Hip fracture | 1 |
| 1. HIV: mother to child transmission | 1 |
| 1. Impacted wisdom teeth | 1 |
| 1. Infantile colic | 1 |
| 1. Infertility associated with endometriosis | 1 |
| 1. Late neurological lyme disease | 1 |
| 1. Leprosy | 1 |
| 1. Low back pain (chronic) | 2 |
| 1. Low back pain and sciatica (acute) | 2 |
| 1. Malaria | 1 |
| 1. Malignant melanoma (non-metastatic) | 1 |
| 1. Nocturnal enuresis | 1 |
| 1. Ocular herpes simplex | 2 |
| 1. Osteoarthritis | 1 |
| 1. Otitis media with effusion | 4 |
| 1. Ovarian cancer | 1 |
| 1. Pelvic inflammatory disease | 1 |
| 1. Perinatal asphyxia | 1 |
| 1. Plantar heel pain and fasciitis | 2 |
| 1. Post-traumatic stress disorder | 2 |
| 1. Postherpetic neuralgia | 3 |
| 1. Premenstrual syndrome | 1 |
| 1. Preterm birth | 4 |
| 1. Primary prevention | 2 |
| 1. Recurrent miscarriage | 4 |
| 1. Reducing pain during blood sampling in infants | 3 |
| 1. Secondary prevention of ischaemic cardiac events | 7 |
| 1. Stomach cancer | 1 |
| 1. Stroke management | 2 |
| 1. Stroke prevention | 4 |
| 1. Tennis elbow | 1 |
| 1. Tinnitus | 1 |
| 1. Trigeminal neuralgia | 1 |
| 1. Upper respiratory tract infection | 1 |
| 1. Urinary tract infection in children | 1 |
| 1. Ventricular tachyarrhythmias (out of hospital cardiac arrests) | 1 |
| 1. Warts | 1 |
| Total | 129 |
