# Supplementary material for: How Often Are Ineffective Interventions Still Used in Clinical Practice? A Cross-Sectional Survey of 6,272 Clinicians in China
Source: PLoS One. 2013 Mar 22;8(3):e52159. doi: 10.1371/journal.pone.0052159 (PMC3606390; doi:10.1371/journal.pone.0052159)
Supplement: Table S3 — Prescription rate of physicians, relative reduction in prescription rate, frequency of use in patients, and relative reduction in frequency of use according to departments where the interventions are mostly used. (DOCX) [file pone.0052159.s006.docx]

Table S3. Prescription rate of clinicians, relative reduction in prescription rate, frequency of use in patients, and relative reduction in frequency of use in patients according to departments where the interventions are mostly used

| Department | Number of clinicians | Number of interventions | Ineffective interventions | | | Effective interventions | | | Rrelative reduction in PR (%)  (e =  100×(c-a)/c) | Relative reduction in FU (%)  (f =  100×(d-b)/d) |
| --- | --- | --- | --- | --- | --- | --- | --- | --- | --- | --- |
|  |  |  | CIT* | Prescription rate (PR) | Frequency of use (FU)±SD  (b) | CIT* | PR | FU±SD  (d) |  |  |
|  |  |  |  | (a) |  |  | (c) |  |  |  |
| Infectious disease | 197 | 7 | 506 | 49.8 | 23.5±31.4 | 261 | 72.0 | 51.8±42.1 | 30.8 | 28.4 |
| Pediatrics | 543 | 14 | 2665 | 44.9 | 24.5±35.2 | 1903 | 55.6 | 32.4±39.1 | 19.2 | 7.8 |
| ENT(ear-nose-throat) | 238 | 6 | 1246 | 70.8 | 43.0±38.5 | 626 | 72.0 | 48.4±42.3 | 1.7 | 5.4 |
| Obstetrics and gynecology | 475 | 28 | 7574 | 55.1 | 31.3±37.2 | 3934 | 77.3 | 52.5±39.4 | 28.7 | 21.2 |
| Orthopedics | 628 | 19 | 7567 | 72.5 | 45.6±39.6 | 4395 | 79.3 | 49.2±38.5 | 8.6 | 3.7 |
| Respiratory | 758 | 5 | 3144 | 74.5 | 31.7±31.0 | 2536 | 91.9 | 57.8±32.8 | 18.9 | 26.1 |
| Psychiatry | 236 | 10 | 1683 | 69.5 | 34.0±33.7 | 579 | 76.3 | 41.3±34.1 | 8.9 | 7.3 |
| Dentistry | 310 | 2 | 373 | 66.8 | 28.8±31.7 | 564 | 61.3 | 42.1±40.8 | -9.0 | 13.3 |
| Neurology | 644 | 10 | 4273 | 51.3 | 24.3±32.7 | 2827 | 87.9 | 62.4±37.2 | 41.6 | 38.1 |
| Neurosurgery | 265 | 3 | 377 | 36.3 | 14.8±26.4 | 141 | 99.3 | 91.4±19.6 | 63.4 | 76.8 |
| Nephrology | 345 | 8 | 1806 | 62.5 | 35.6±37.6 | 583 | 57.6 | 31.9±37.5 | -8.5 | -3.7 |
| General surgery | 551 | 1 | 544 | 63.6 | 12.5±15.9 | 551 | 98.4 | 82.4±25.3 | 35.4 | 69.9 |
| Gastroenterology | 590 | 3 | 1748 | 91.3 | 64.3±35.1 | 592 | 89.4 | 56.9±37.0 | -2.1 | -7.4 |
| Cardiology | 968 | 26 | 17587 | 50.0 | 24±32.9 | 6393 | 87.8 | 67.7±37.8 | 43.1 | 43.4 |
| Sexually transmitted diseases and dermatology | 189 | 9 | 1014 | 62.0 | 33.2±35.8 | 790 | 92.9 | 69.8±35.1 | 33.3 | 36.6 |
| Ophthalmology | 225 | 4 | 751 | 41.3 | 19.4±31.1 | 383 | 64.8 | 51.0±45.7 | 36.3 | 31.7 |
| Oncology | 234 | 3 | 410 | 79.0 | 37.3±35.2 | 407 | 92.1 | 75.0±34.2 | 14.2 | 37.7 |

* CIT=clinician-intervention times
