# Supplementary material for: How Often Are Ineffective Interventions Still Used in Clinical Practice? A Cross-Sectional Survey of 6,272 Clinicians in China
Source: PLoS One. 2013 Mar 22;8(3):e52159. doi: 10.1371/journal.pone.0052159 (PMC3606390; doi:10.1371/journal.pone.0052159)
Supplement: Text S1 — An example question asked for effective and ineffective interventions. (DOCX) [file pone.0052159.s007.docx]

**Text S1. An example question asked for effective and ineffective interventions**

1. Have you ever treated patients with acute low back pain and sciatica to reduce pain or disability? (The first sub-question)

| ① Yes | Please answer question 1.1 and 1.2 |
| --- | --- |
| ② No | Please go to question 2 |

| 1.1 In the past 12 months, have you ever used bed rest [an ineffective therapy] to reduce pain or disability in patients with acute low back pain or sciatica? (The second sub-question for an ineffective intervention.)  ① Yes  How often did you use bed rest to reduce pain or disability in every 10 patients with acute low back pain or sciatica you treated in the past 12 months?  %  ② No |
| --- |
| 1.2 In the past 12 months, had you ever used analgesics [the matched effective therapy] to reduce pain or disability in patients with acute low back pain or sciatica? (The second sub-question for an effective intervention.)  ① Yes  How often did you use analgesics to reduce pain or disability in patients in every 10 patients with acute low back pain or sciatica you treated in the past 12 months?  %  ② No |
