# Supplementary material for: How Often Are Ineffective Interventions Still Used in Clinical Practice? A Cross-Sectional Survey of 6,272 Clinicians in China
Source: PLoS One. 2013 Mar 22;8(3):e52159. doi: 10.1371/journal.pone.0052159 (PMC3606390; doi:10.1371/journal.pone.0052159)
Supplement: Text S2 — The letter to the surveyed clinicians. (DOCX) [file pone.0052159.s008.docx]

**Text S2. The Letter to the Surveyed Clinicians**

Dear doctor,

We are researchers from the Peking University Center for Evidence Based Medicine.

We are conducting a survey about the use of clinical interventions in the past 12 months. If you have not seen any patients in the past 12 months as your clinical work, you are not eligible for this study. We thank you for your attention.

Otherwise, we would appreciate very much if you could spare some of your valuable time with us and participate in this survey, which takes about 10 minutes all together. In this survey, we will not ask you to put down your real name. Your answers to the questions will be kept private and confidential and will be used only for the research purposes and not be disclosed to or used for any other purposes and by anyone beyond the core members of the research team without your prior consent.

Taking part in this study is completely voluntary. If you decide to take part in the study, you are free to withdraw from it anytime or skip any questions that you do not want to answer. We assure you that any of your decisions about participating in the study will be respected and will not cause any inconvenience to you.

For any further inquiries, you may contact Ms Luo Xiao-min or Prof. Tang Jin-ling on 010-82801108 at Peking University Center for Evidence Based Medicine.

Peking University Center for Evidence Based Medicine

June 2005
