# Supplementary material for: How Often Are Ineffective Interventions Still Used in Clinical Practice? A Cross-Sectional Survey of 6,272 Clinicians in China
Source: PLoS One. 2013 Mar 22;8(3):e52159. doi: 10.1371/journal.pone.0052159 (PMC3606390; doi:10.1371/journal.pone.0052159)
Supplement: Text S3 — A survey on the use of some clinical interventions in China. (DOCX) [file pone.0052159.s009.docx]

**Text S3.** A **Survey on the Use of Some Clinical Interventions in China**

**By Peking University EBM centre**

This study is designed for clinicians of western medicine. If your routine work is not clinical in western medicine but in such areas as health policy, basic medical research, pharmacy, traditional Chinese medicine, disease control and prevention, you are not invited to participate in the survey.

Dear doctors,

We welcome you and invite you to participate in this survey!

This study is to investigate the variation in use of some clinical interventions in the country and the reasons for the variation so as to provide information for further improving health services and for developing evidence-based medicine in the country.

The questions we are to ask are supposed to be about how you ACTUALLY treat your patients rather your opinions or recommendations. There is no right or wrong answers to any of the questions; we only hope to compare the practice among doctors and in areas.

All the data and information you provide will be used for the research purposes only and will be reported only in a summary or average manner. Therefore, you data will be kept private and confidential and without your prior consent will not be disclosed to, or used for any other purposes by, anyone beyond the core members of the research team. Even data analysts will not be able to see your personal data including your email address and ID if you provide them.

To express our thanks for your contribution, after the survey is completed, we will be arranging a luck draw among all the participants. Please kindly leave your email and ID if you wish to join the luck draw. The prizes are:

One 1st prize: gifts worth $5,000 RMB

Five 2nd prizes: gifts worth $1,000 RMB

Fifty 3rd prizes: one of three books on evidence-based medicine in Chinese

If you have any further inquiries about this survey, please contact Peking University EBM centre at:

4th Floor, School of Public Health Building

Peking University Health Science Centre

No. 38, College Road, Haidian District

Beijing Post Code: 100083

Contact person: Ms Luo Xiao-min

Tel: 010-8280 1108

Fax: 010-8207 7775

Email: pkuebm@bjmu.edu.cn

Website: http://pkuebm.bjmu.cn/

We sincerely thank you for your support and participation.

Center for Evidence Based Medicine, Peking University

November 2006
